# The Lived Experience of Multiple Sclerosis Relapse: How Adults with Multiple Sclerosis Processed Their Relapse Experience and Evaluated Their Need for Postrelapse Care

**DOI:** 10.1155/2015/351416

**Published:** 2015-05-13

**Authors:** Miho Asano, Karli Hawken, Merrill Turpin, Abby Eitzen, Marcia Finlayson

**Affiliations:** ^1^School of Rehabilitation Therapy, Queen's University, 31 George Street, Kingston, ON, Canada K7L 3N6; ^2^School of Health and Rehabilitation Sciences, University of Queensland, Brisbane, QLD, Australia

## Abstract

*Background*. Multiple sclerosis (MS) relapses can take a toll on individuals' health and quality of life. Given such consequences of relapses, postrelapse care beyond pharmacological approaches may play an important role in recovery. Nevertheless, how individuals with MS process their relapse experience and manage the consequences is still uncertain. *Purpose*. We conducted a qualitative study to understand relapse experiences and postrelapse care need from perspectives of adults with MS and identify relapse management patterns. *Methods*. We interviewed 17 adults with MS. *Results*. By examining combinations of three categories related to relapse experience, we identified four relapse management patterns: (i) *Active Relapse Manager*, (ii) *Early-Stage Proactive Relapse Monitor*, (iii) *Adapted Passive Relapse Manager*, and (iv) *Passive Relapse Monitor*. The relapse management patterns appear to associate strongly with the appraisal of the experience. *Conclusions*. The results of this study suggest the importance of understanding each patient beyond their functional limitations and the potential need for multidisciplinary postrelapse care which goes past restoring functional limitations at the acute phase. Future research to further understand the relapse management process at all stages of the healthcare continuum is a crucial step toward developing strategies to advance the current postrelapse care and to facilitate optimal recovery.

## 1. Introduction

Multiple sclerosis (MS) is a progressive and incurable neurological disease. It is estimated that over two million people worldwide are living with MS [1, 2]. The prevalence of individuals with MS in North America (i.e., Canada and the United States (US)) is reported to be one of the highest in the world [1, 2].

Up to 85% of individuals with MS are initially diagnosed with relapsing-remitting MS (RRMS) [3]. Most individuals who are diagnosed with RRMS experience periodic relapses. Relapses are characterized by episodes of focal neurological disturbance [4] and often result in the appearance of new symptoms or worsening of existing symptoms lasting from 24 hours to several weeks. Depending on the characteristics of a relapse (e.g., type, severity, and duration of a relapse) which may vary for each occurrence, it can take a toll on individuals' health, well-being, and quality of life.

Relapses are commonly treated by steroids. Steroids may reduce the acute inflammation, but they do not address the challenges of managing symptoms and functional disabilities during and/or after the acute phase. Up to 58% of individuals with MS report a measurable and sustained effect of relapses on disability [5]. These residual disabilities can lead to activity limitations and participation restrictions in daily life. A recent survey study revealed that, compared to individuals with MS in remission, those who experienced a relapse reported significantly worse physical functioning and mental health [6].

Given the known consequences of relapses, postrelapse care beyond pharmacological approaches such as rehabilitation services may play an important role in recovery. Nevertheless, how individuals with MS process their relapse experience and manage consequences of their relapse is still uncertain [7]. The aims of the study were to (1) understand relapse experiences and postrelapse care need from perspectives of adults with MS and (2) identify relapse management patterns.

## 2. Methods

The study aims were addressed by a cross-sectional qualitative study informed by interpretive description [8, 9]. Thorne at al. (1997) state that “the foundation of interpretive description is the smaller scale qualitative investigation of a clinical phenomenon of interest to the discipline for the purpose of capturing categories and patterns within subjective perceptions and generating an interpretive description capable of informing clinical understanding [8].” This approach was selected because the goal of the study was to generate new knowledge to inform the current postrelapse care in MS. This study was conducted in Chicago, IL (USA) and Kingston, ON (Canada). Prior to commencing, the study was approved by the relevant university ethics committees in both locations.

### 2.1. Participants

To be eligible, individuals had to be 18 years of age or older; self-report a diagnosis of MS; self-report a physician-verified MS relapse within the past six months of their interview; and live within 30 miles of downtown Chicago or Kingston. Individuals were excluded if they were unable to follow, complete, or physically tolerate an interview of approximately one hour in English.

### 2.2. Recruitment

Information flyers, brochures, and invitation letters were distributed to potential participants through the local MS clinics in Chicago and Kingston by collaborating healthcare professionals (i.e., occupational and physical therapists, neurologists, and nurses). Individuals interested in participating in the study were asked to call the research office. A trained researcher answered the calls, described the study, responded to questions, and screened them for their eligibility. Once eligibility was confirmed, individuals scheduled a meeting with the researcher for the informed consent and interview.

### 2.3. Interview

Each participant completed one semistructured interview that lasted an average of 107 minutes (SD = 32; range = 55–150 minutes). Most participants chose to have their interview conducted in their own home (*n* = 13/17, 76%). The interview guide focused on gathering information related to the participants' experiences with their most recent relapse. Sample questions included the following: How would you describe your most recent relapse experience? How did your relapse affect you, your daily routine and activity? and What things do you or did you do to manage your most recent relapse? All the interviews were conducted in person and digitally recorded with permission. At the end of the interview, participants completed self-reported questionnaires about their ambulatory disability (Patient Determined Disease Steps (PDDS) [10, 11]), MS (e.g., years since diagnosis, time since the most recent relapse, and Multiple Sclerosis Impact Scale 29 (MSIS 29) [12]), and sociodemographic information (e.g., age, sex, and employment status).

### 2.4. Data Management and Analysis

Approximately two weeks after the interview, 16 participants (94%) completed a member check procedure. One participant was unable to complete the process due to rehospitalization. A summary of their individual interview was first mailed to each participant. Then a researcher called the participants and conducted the member check. Feedback on the summary was obtained from participants to confirm the accuracy of the interview and to enhance the dependability of the data [13]. All the interviews were transcribed verbatim and reviewed against the digital recordings for accuracy. Three members of the team reviewed the transcriptions several times before initiating the coding process. Once coding began, the team met biweekly to discuss quotes and emerging categories. The team refined the categories until they reached consensus. Each category in this study represents a major construct related to MS relapse experience.

Upon reaching consensus, three members of the team coded the data using qualitative data-analysis software (ATLAS.ti). Next, a member reviewed potential combinations of categories to identify relapse management patterns and created a matrix (in which participants were grouped based on combinations of identified categories). Once the matrix was completed, two different members independently confirmed the groupings and its contents. Identifying unique combinations of the categories allowed the team to assess the presence and the characteristics of relapse management patterns. The quantitative data were analyzed descriptively (e.g., *n*, %, mean and standard deviation) to illustrate the characteristics of the participants.

## 3. Results

### 3.1. Participants and Their Most Recent Relapse

Seven individuals from Illinois and ten individuals from Ontario participated in the study. On average, participants were interviewed within 2.5 months (±2 SD; range = 1 week–6 months) of the occurrence of their most recent relapse. The mean age was 42 years (±12 SD; range = 26–69 years). The majority were women (*n* = 16/17, 94%).

Participants were diagnosed with MS an average of 11 years prior to the interview (±8 SD; range = 1 week–28 years). The median PDDS score was 3 (ranged from 0 to 7), which means that 50% of the participants reported mild to moderate ambulatory disability that interfered with their activities. Approximately 30% of the participants were using a cane or a walker at the time of their interview.

The median MSIS-29 physical impact score was 51 (ranged from 0.0 to 92.5) and psychological impact score was 50 (ranged from 0.0 to 94.4). The scores for the MSIS-29 physical and psychological impact range from 0 to 100. Higher scores indicate worse self-reported health.

Eight participants (47%) reported the use of steroids and five participants (29%) were hospitalized due to their most recent relapse. Seven participants (41%) used postrelapse rehabilitation services, but, of those, three participants only received a single session (mostly for the assessment).

### 3.2. Categories

Three categories were identified in the data:* Description of Relapse Experience*,* Interpretation of Relapse Experience*, and* Perceived Need for Postrelapse Care* (Table 1 summarizes descriptions of categories and corresponding quotes). Whether it was a new symptom/limitation or a worsening of a preexisting symptom/limitation, all of the participants reported at least one symptom/limitation that they experienced due to the recent relapse.

These symptoms/limitations led to the first category of the analysis—*Description of Relapse Experience*—which included three subcategories: Impact of Relapse on* Physical*,* Cognitive*, and* Emotional Health*. Largely based on how the symptoms/limitations affected the participants' daily routine and quality of life, they interpreted their experience in one of three distinctive ways. The second category of the analysis emerged—*Interpretation of Relapse Experience*—which included three subcategories: the most recent relapse as a* Minor Event* with no impact, a* Manageable Event* with some impact, or a* Major Event* with severe impact on daily life. The third category of the analysis—*Perceived Need for Postrelapse Care*—arose and also included three subcategories: Participants perceived postrelapse care as* Necessary*,* Unnecessary*, or* Undetermined*.

### 3.3. Patterns

Examining combinations of these categories, we identified four relapse management patterns—how participants processed their relapse experience and evaluated their need for postrelapse care: (i)* Active Relapse Manager*, (ii)* Early-Stage Proactive Relapse Monitor*, (iii)* Adapted Passive Relapse Manager*, and (iv)* Passive Relapse Monitor*. The following section provides descriptions of the four patterns (see Table 2 for more details).

#### 3.3.1. Active Relapse Manager

Participants placed in this pattern were mostly at the peak of their career and family development. They reported experiencing severe symptoms/limitations during and/or after the relapse. At the time of their interview, they acknowledged that these symptoms/limitations were preventing them from performing necessary/desired activities in their daily life (e.g., inability to continue their employment, to take care of their family, and to perform basic activities of daily living). The participants perceived the relapse as a negative and significant event and clearly expressed their need for postrelapse care. A participant in her early 30s who was on disability leave at the time of her interview stated that:

*All right, well how it affected my daily life was completely… I had to teach myself to eat with my wrong hand… Those things are truly earth-shattering. I keep on referring to myself - I live the life of a retired person now, kind of crippled, kind of available 24-hours a day but sleeping half of that time… I continually say I have a problem with this…I need help now.*

#### 3.3.2. Early-Stage Proactive Relapse Monitor

Similarly, participants placed in this pattern also reported experiencing severe symptoms/limitations that prevented them from performing necessary and/or desired activities in their daily life. The main difference between the two patterns was that the participants in the* Early-Stage Proactive Relapse Monitor *pattern were interviewed within two weeks of the occurrence of a relapse. These participants perceived the relapse as a negative and significant event, but possibly as a temporal matter. At the time of their interview, they were monitoring their symptoms/limitations while waiting for a scheduled appointment with their neurologist to discuss their recovery process and treatment options. They were uncertain about their need for current care while acknowledging their potential need for future care. A participant in her early 50s on disability leave who was interviewed within a week of her relapse stated that:

*I've experienced them before, but this was probably a more severe one… I do not have a daily activity, really. I cannot do anything… I'm angry that I cannot do the things that I used to be able to do… Because I do not really think there's anything that can be done about it…it's just been sort of like a waiting game. It's just like to wait it out… It's just I have to see if it will come back.*

#### 3.3.3. Adapted Passive Relapse Manager

Participants in the third pattern provided descriptions of symptoms/limitations, similar to the participants in the two aforementioned patterns. What differentiates the participants in this pattern from the first two patterns is that they perceived the relapse as a manageable event. The participants in this pattern were able to continue performing necessary and/or desired activities in their daily life even with the symptoms/limitations due to the relapse. A common explanation as to why these participants found their relapse manageable was that they had similar experiences with the previous relapses and had already altered their daily routines in the past. They felt that nothing could improve their current condition and expressed no need for postrelapse care. A participant in her 30s with over 10 years of MS experiences who was working full time at the time of her interview stated that:

*It's not pain; it's ache, and it's uncomfortable…So it wasn't anything catastrophic like before… It was just that I noticed when I was working, and things weren't as easy as they used to be; not easy, but it was increasingly more difficult…you know, because it's been so long with the same problems, and then noticing that it's really gotten more difficult… like, I can still do it…it wouldn't be life-changing, this, no, because I haven't changed my life. It just makes it more difficult…I think nothing else can be done….*

#### 3.3.4. Passive Relapse Monitor

Unlike other participants in the study, participants placed in this pattern reported experiencing a short-term acute relapse or a minor relapse without significant residual disabilities. All the participants in this pattern were able to continue their daily routine during and/or after the relapse. As a result, they perceived the relapse as a minor event and expressed no need for postrelapse care. A participant, just turning 60, who experienced only two relapses over 10 years since her diagnosis stated that:

*Well, it has not been very dramatic, or it has not really altered what I can and cannot do… Like I say, it's just been so mild. Other than the (mild) optic neuritis, it just – most of the time I forget I even have it. I do not think of it… I know that it's probably not long term, and I feel confident that it will clear up on its own… So I'm not sure that there would be anything that I could do, other than just try to keep my stress level down.*

**Figure 1:**
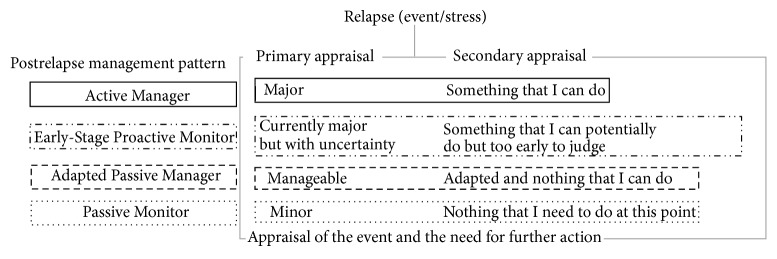
Relapse experience and management appraisal schematization (based on Lazarus and Folkman's transactional model of stress, coping, and adaptation).

Once these patterns were determined, we recognized that they are well aligned with Lazarus and Folkman's transactional model of stress, coping, and adaptation [14], specifically, its phase of the primary and secondary appraisal of an event. According to the model, at the primary appraisal phase, individuals evaluate their situation by posing a question such as “what does this event mean to me?” At the secondary appraisal phase, individuals evaluate their coping resources options by asking a question such as “what can I do about the event?” [14–16]. A link between the primary and secondary appraisal of an event (i.e., the interpretation of the relapse experience and the perceived need for postrelapse care) is shown in the additional examples drawn from our data (see Figure 1 for a graphical presentation of the data):

 At the primary phase,* active relapse managers* identify the relapse as a negative and significant event. At the secondary phase, they express their belief that there is something that they can do to resolve the challenges associated with or caused by the event and there is a need for postrelapse care.
 
*Early-stage proactive relapse monitors* also identify the relapse as a negative and significant event at the primary phase. However, the secondary appraisal—their belief of whether or not there is something that they can do to resolve the challenges associated with or caused by the event—is undetermined due to a potential temporal factor (i.e., early-stage proactive monitors were interviewed within two weeks of the relapse incidence). Reappraisal of the event is likely to occur based on the recovery process and/or new information that may arise in the future.
 Despite the fact that* adapted passive relapse managers* may experience substantial functional limitations due to a relapse, at the primary phase they identify the relapse as a nonsignificant event. At the secondary phase, they express their belief that there is nothing that they can do to resolve the challenges associated with or caused by the event and therefore there is no need for postrelapse care. Temporal, personal, and environment factors can all affect both phases of the appraisal.
 
*Passive relapse monitors*, unlike others, experience a short-term acute and/or a minor relapse with no residual disabilities. Thus they identify the relapse as a nonsignificant event at the primary phase. Mainly, due to the nature of the relapse, at the secondary phase, they express their belief that there is nothing that they need to resolve for this particular event and therefore no need for postrelapse care.

## 4. Discussion

All the participants in our study reported experiencing new or worsening symptoms and/or limitations (e.g., problems with walking, memory, or fatigue) due to the most recent relapse. The findings confirm the diverse nature of the symptoms and limitations that people with MS may experience during and after a relapse.

Four unique and distinct categories of relapse management patterns were identified in this study. The interpretation of the relapse experience appears to closely associate with the perceived need for postrelapse care to form these patterns. Factors such as participants' capacity to perform necessary and/or desired activities in their daily life, duration of the relapse, and/or past experiences with relapses seem to weigh in on their relapse interpretation and perceived need for postrelapse care. Types or severities of symptoms and/or limitations, on the other hand, appear to play a less important role when considered on their own.

Agreeing with Lazarus and Folkman [14], our results suggest that coping with and adapting to an event—a relapse—is not a static process; it is a transactional process that commonly depends on many factors and changes over time. The process of appraising a relapse goes beyond the quantification of functional limitations. This process can be influenced by combinations of temporal, personal, and environmental factors (e.g., the timing of considering care attainment, the belief about specific care, or the presence of health insurance coverage) that are often unique to an individual and even to each relapse. This was a cross-sectional study without follow-up data collection. Therefore, we are unable to confirm the effect of mediating and temporal factors as well as the potential causal associations that may occur in the transactional process of the relapse experience. This may be considered as a limitation of the study. A future longitudinal study may prove valuable for deepening our understanding of the relapse experience and management process.

Given the descriptions of the relapse experience, healthcare professionals may easily assume a key role in postrelapse care (e.g., rehabilitation) to facilitate optimal recovery. Our results however suggest that the appraisal of relapse experience leading to such care attainment depends on individuals and their circumstances. We acknowledge that many reasons unexamined in depth in this study can influence this appraisal. It was intriguing to find that many participants shared their belief that there was nothing that they could do to improve or regain their impaired/lost function. Such individual appraisals may prove inaccurate when measured by a third party (e.g., rehabilitation specialists) and can be considered as a barrier to attaining appropriate postrelapse care.

The outcome of appraising an event, along with other factors, plays a significant role in how individuals determine and execute coping or adaptation strategies [17]. According to Lazarus and Folkman [14], interventions aimed at manipulating factors attributing to the process of coping or adaptation, when steered successfully, can modify one's appraisal of an event. Understanding factors attributing to the process of appraising relapses could provide healthcare professionals and researchers with useful insights as to what adults with MS take into account while making care decisions. This is however out of the scope of our current analysis. Our future study will analyze the existing data in depth and address this topic.

A total of 17 adults who recently experienced a relapse completed their semistructured interview, mostly at their home. We were unable to objectively measure functional limitations due to the relapse or confirm the exact date of the relapse incidence (e.g., physical exams, performance-based tests, and MRI). A lack of such data may be considered as another limitation of this study. Due to the sample size and sample characteristics (e.g., gender ratio and geographical locations), the findings may not be applicable to many others with MS. A quantitative study to survey a larger number of people with MS about their relapse experience, postrelapse care, and rehabilitation services may be considered as a necessary future step to further understand the challenge of and the need for the postrelapse care in MS.

The number of people who are diagnosed with chronic diseases continues to grow rapidly [18, 19]. Therefore, coping research to manage chronic diseases such as MS has become an important area of healthcare in the past two decades [19]. A recent comprehensive review of psychological correlates of adjustment among individuals with MS [20] identified 44 studies that investigated the topic of stress and coping; however, only 3 out of the 44 studies examined a link between cognitive appraisals of MS related stressors and adjustments [21–23]. To our knowledge, this study is one of the first to investigate the lived experience of MS relapse in the area of coping research and therefore makes unique contributions to the existing body of evidence.

## 5. Conclusion

MS disease modifying agents have reduced the rates of relapses and slowed down the progression in the past two decades. Most people diagnosed with RRMS still continue to experience periodic relapses [24] that can lead to residual disabilities, reduced productivity, or diminished QoL [5, 6]. Steroids, commonly prescribed to treat acute relapses, may reduce the inflammation due to relapses and shorten their duration; however, they do not aid people with MS to manage their disabilities easily during and/or after the acute phase [25]. Many participants in this study reported functional limitations and residual disabilities due to a relapse and therefore they were unable to continue performing daily activities of their choice. There are four relapse management patterns that we identified in this study. Primary and secondary appraisal of a relapse—the interpretation of the relapse experience and the perceived need for postrelapse care—appear to be key factors that determine these patterns. Many factors, unassessed in depth in this study, can influence these patterns as well. However, characteristics of symptoms and/or limitations due to a relapse appear to be less of importance on their own to the individuals with MS in their relapse appraisal process.

The results of this study suggest the importance of understanding each patient beyond their functional limitations and the potential need for multidisciplinary postrelapse care which goes past restoring functional limitations at the acute phase. Future research to further understand the relapse management process at all stages of the healthcare continuum is a crucial step toward developing strategies to advance the current postrelapse care and to facilitate optimal recovery.

## Figures and Tables

**Table 1:** Basic descriptions of categories and corresponding quotes.

Main category	Subcategory	Definition	Quotes
Description of Relapse Experience	Impact on Physical Health	Descriptions related to physical limitations caused by the most recent relapse	“Excruciating pain through my arms, like that burning, and just feeling – the arms feel really heavy, like I can't use them, extremely weak.”
	Impact on Cognitive Health	Descriptions related to cognitive limitations caused by the most recent relapse	“It's just reading sentences or certain things that I will have to keep reading and keep reading and keep reading to completely understand it … something as simple as getting directions to my husband's job that I know that he's been working there for 20 years. I know how to get there, but I could not – I just couldn't understand it. I couldn't understand what he was saying, so I had to write down verbatim …”
	Impact on Emotional Health	Descriptions related to one's emotional health being affected and/or changed by the most recent relapse	“This one [relapse], I think, more affected my pride, and it affected me more emotionally because I wasn't able to function as me.”

Interpretation of Relapse Experience	Minor Event with No Impact	Making reference to having no consequences to or no changes in themselves or their lives as a result of the most recent relapse	“Like I say, it's [relapse] just been so mild. Other than the optic neuritis, it just – most of the time I forget I even have it. I don't think of it.”
	Manageable Event with Some Impact	Making reference to having manageable consequences to or changes in themselves or their lives as a result of the most recent relapse	“…I'm still able to do them [daily activities]; it just takes me longer to do them…” “It [relapse] was easy for me because I felt as though I had already went through this experience right before, and I basically knew what to expect.”
	Major Event with Severe Impact	Making reference to having severe consequences to or major changes in themselves or their lives as a result of the most recent relapse	“Well, how it [relapse] affected my daily life was completely. If I took my daily life before, it was not possible … I had to move out of my house for two-and-a-half months. I couldn't live here. Everything was different.”

Perceived Need for Postrelapse Care	Necessary	Making reference to requiring/desiring healthcare services to manage the most recent relapse	“I continually say, I have a problem with this [inability to self-care]. And everyone said, just give it time. No, I need help now.”
	Unnecessary	Making reference to not requiring/desiring any healthcare services to manage the most recent relapse	“…the symptoms of optic neuritis, but I really don't think there's an awful lot you can do better because it's not the eyeball. Because it's the pathway up to the brain.”
	Undetermined	Making reference to undetermined needs/desires for healthcare services to manage the most recent relapse	“…they say that it [vision] will usually come back itself over time, I decided just to try to wait it out this time and see what happens.”

**Table 2:** Basic descriptions of four patterns.

	Active Relapse Manager	Early-Stage Proactive Relapse Monitor	Adapted Passive Relapse Manager	Passive Relapse Monitor
*Personal profile *				
Age	26 to 56 years	33 and 50 years	36 to 69 years	33, 34, and 60 years
Time since diagnosis	3 months to 22 years	8 and 12 years	10 to 28 years	4 to 11 years
Time since relapse	1 month to 5 months	<2 weeks	1 week to 6 months	2 to 3 months

*Description of relapse experience *				
Examples: symptoms or limitations	(i) Problems with balance, mobility, memory, expression, fatigue, anxiety, depression, and fear (ii) Inability to self-care, to walk, to read, to think, to remember, to drive, and to run errands in their community	(i) Problems with vision, memory, fatigue, pain, mobility, depression, fear, and anger (ii) Inability to manage basic house chores, to read, to think, to remember, and to drive to work	(i) Presence of pain, fatigue, numbness in hands or legs, heaviness in legs, problems with memory, and word searching	(i) Presence of acute short-term pain without residual disabilities, minor problems with vision, or numbness between toes
Notion about daily routines	(i) Daily routine significantly affected (ii) Unable to work and to perform necessary or desired activities in their daily life (e.g., socializing, taking care of house chores, pursuing regular hobbies, and leisure activities)	(i) Daily routines altered significantly (ii) Unable to work and to perform necessary or desired activities in their daily life (e.g., taking care of young children, family members and performing basic house chores)	(i) Daily routine was not altered significantly due to the most recent relapse (ii) Able to manage and continue performing necessary or desired activities of their daily life	(i) Daily routine was not altered (ii) Able to manage and continue performing necessary or desired activities of their daily life

Interpretation of relapse experience	Severe	Severe	Manageable	Minor (including a short-term acute relapse with no residual disability)

Perception of postrelapse care need	Necessary	Uncertain (i) Possibly due to being at the early stage of a relapse (ii) Presence of self-monitoring the progress of the relapse	Unnecessary (i) Possibly due to the belief about postrelapse care and the disease progression	Unnecessary (i) Possibly due to a lack of residual disabilities

Summary	(i) Participants in this pattern were mostly at the peak of their career and family development phase (ii) Symptoms and/or limitations due to the relapse were preventing the participants in this pattern from performing necessary or desired activities in their daily life (iii) These participants had to make considerable changes in their daily routine to cope with their challenges and/or disabilities that occurred during and after relapse	(i) Participants in this pattern were at the peak of their career and family development phase (ii) Symptoms and limitations due to the relapse were preventing participants in this pattern from performing necessary or desired activities in their daily life (iii) These participants interpreted the effect of the relapse as negative and severe; however, they were at the early stage of their relapse and expressed their uncertainty as to whether or not they needed the postrelapse care	(i) Some of the participants in this pattern were at the peak of their career and family development and others were in their retirement (ii) Type or severity of symptoms/limitations due to the relapse did not affect how the participants in this pattern interpreted their relapse experience (iii) The participants interpreted their relapse experience as manageable possibly due to their experiences with the past relapses and belief about available care being ineffective	(i) Some of the participants in this pattern were at the peak of their career and family development and others were in their retirement (ii) Participants in this pattern experienced minor or short-term acute symptoms/limitation without residual disabilities (iii) These participants were able to continue performing necessary or desired activities in their daily life without altering their daily routine; thus, they expressed no need for postrelapse care
